# The Impact of Nutrition Labelling on Customer Buying Intention and Behaviours in Fast Food Operations: Some Implications for Public Health

**DOI:** 10.3390/ijerph19127122

**Published:** 2022-06-10

**Authors:** Abu Elnasr E. Sobaih, Ahmed Sh. Abdelaziz

**Affiliations:** 1 Management Department, College of Business Administration, King Faisal University, Al-Ahsa 31982, Saudi Arabia; 2 Hotel Management Department, Faculty of Tourism and Hotel Management, Helwan University, Cairo 12612, Egypt

**Keywords:** nutrition labelling, buying intention, buying behaviour, fast food operations (FFOs), theory of planned behaviour, healthy food choice

## Abstract

This research examines customers’ intention to buy depending on their use of nutrition labelling (NL) in fast food operations (FFOs) and their intention to visit and recommend these FFOs with nutrition-labelled menus. The research model draws on the theory of planned behaviour (TPB) to examine customers’ intentions to buy from nutrition-labelled menus and their behaviour of visiting and recommending to others FFOs with nutrition-labelled menus. To achieve this purpose, a self-administrated questionnaire was distributed to and collected from a random sample of customers at FFOs in Greater Cairo, Egypt, i.e., McDonald’s and Subway. The results from the structural equation modelling (SEM) using AMOS software indicated positive and direct significant paths from the constructs of the TPB, except for customers’ attitude, to customer intention to buy nutrition-labelled menu items. The results also showed a positive significant impact of customers’ intention on their behaviour of visiting and recommending FFOs featuring nutrition-labelled menus. The findings showed that there is an awaking of nutritional awareness among fast-food customers and that providing nutritional information on fast-food menus will affect their purchasing intention in the future by encouraging them to make healthy food choices. Theoretical implications for scholars and managerial implications for FFOs, especially in relation to public health in general and healthy food choices in particular, are explained and discussed.

## 1. Introduction

Dining out has become an important part of the modern lifestyle; thus, there is an increasing trend of eating away from home to keep up with the rapid pace of working life. This is reflected in the growth of fast-food operations (FFOs) worldwide. However, studies often link eating out with obesity and the perceptions that FFOs often provide unhealthy food [1]. This affects the image of FFOs as unhealthy food choice for customers. In response to this, some international FFOs chains, mainly American brands, e.g., McDonald’s, responded by providing nutrition information (NI) on their website and labels containing nutritional information about each served meal on the menu to convince their customers that they are providing healthy food options.

The US Food and Drug Administration (FDA) enacted the Nutrition Labelling and Education Act (NLEA) in 1990, which encouraged a fast-increasing area of research. In 2010, the Patient Protection and Affordable Care Act (ACA) contained a menu calorie labelling mandate, making it one of the first federal-level obesity therapies aimed at the entire community. The FDA, on the other hand, repeatedly postponed the publication of its final regulation until May 2018 [2]. Regulations aimed at maintaining customers’ public health and reducing obesity by giving appropriate nutrition information at the time of purchase as a tool to encourage better food choices have been aimed at the restaurant business [3]. As the cost of these menu nutritional labelling standards is high [4], the impact of nutrition labelling on consumer food choice needs to be clearly demonstrated [5]. The giving of nutritional information at the point-of-purchase menu items is referred to as menu labelling. Total calories, fat calories, saturated fat calories, cholesterol, salt, total and complex carbs, sugars, dietary fibre and protein are all included [6].

Recent studies have been conducted to determine the impacts of disclosing menu item nutrition information on customers’ product assessments, purchase intentions and behaviour [7,8]. A recent study conducted by Aitken et al. [9] on the benefits of menu labelling of organic foods suggested improving labelling to have more actionable information, such as the health, environmental and societal benefits of products. Additionally, the same study showed that consumers’ perceived behavioural control needs to be enhanced to their strengthen intentions to purchase food with organic labels. The existing research on the effectiveness of menu labelling has shown different results. In FFOs providing information on the menu board, it was found that it enhanced consumer awareness [10]. Moreover, numerous different studies have found a decrease in the numbers of calories ordered and consumed after menu labelling [11,12,13]. However, other studies have found only a slight change in the number of calories ordered after menu labelling [14,15]. Another study [16] found that calorie labels reduced the number of calories ordered for some items, but not all of the restaurant items were assessed to ensure the generalizability of this result.

This research examines the influence of nutrition labelling on customers’ buying intention in FFOs and their behaviour of visiting and recommending to other customers those items with nutrition labelling and information. For this purpose, the research adopted the theory of planned behaviour (TPB) to properly understand and examine this relationship. The TPB framework has been applied extensively [(see for example 17–20)] to predict human intention and behaviour. For example, the theory was adopted to predict food consumption decisions [17], intentions to purchase local food products [18] and customer intention and behaviour towards food waste [19]. Shin et al., [20] adopted the TPB to explore and examine customer intention and behaviour towards organic menus in restaurants and gained a better understanding of the determinants of customer intention and behaviours regarding organic menu. Despite the appropriateness of the TPB for predicting customers’ intention and behaviour, to the best of the researchers’ knowledge, there is no published research examining customer intention to buy from FFOs featuring nutrition-labelled menus, especially in non-western countries such as Egypt. This research is among the first attempts at drawing on the TPB for better understanding customer buying intention and behaviour regarding items with nutrition labelling in FFOs. Specifically, the research addresses the implications of these nutrition labels on customers’ healthy choices, which in turn affects the public health.

To achieve the research purpose highlighted above, the current article will be structured as follows. The next section (Section 2) reviews the related studies and discusses the research conceptual model. Section 3 presents the research methodology, especially the data collection methods from the research sample and the data analysis techniques. This section also discusses the research instruments adopted for the data collection. Section 4 presents the results of the research. This section ends with presenting the research structural model. Section 5 discusses the results and compares them with findings from previous studies. It also highlights the theoretical and practical (mainly managerial) research implications. Section 6 presents the research conclusion. The last section (Section 7) discusses the major limitations of the study and highlights opportunities for future research.

## 2. Review of the Literature

### 2.1. Using Nutrition Labelling in Restaurants

Menu labelling provides customers with relevant information about the nutrient content of food items, at the point of purchase, to make customers capable of choosing nutritionally suitable food [21]. Studies [22,23] have confirmed that customers want menu labelling to be available on restaurant menus. Moreover, these studies also confirmed that customers argue that if they see this information, they will make healthier menu choices. The previous studies focused on determining customers’ desire for nutritional information in restaurants, determining what type of nutritional information they specifically want and understanding the formats and methods of presenting nutritional information in restaurants. The basic concept is providing nutritional information that many people do not easy access to in a readily available manner at the point of consumption as well as preventing obesity. With nutrition information, people will become more inclined to use it and make healthier choices [24]. Customers expect restaurants including FFOs to provide nutritional information on the menu, including calorie, sugar, protein, carbohydrate and fat content [25]. The provision of nutritional information on restaurant menus has grown in popularity, as has the number of customers concerned about this issue [23]. Without calorie labelling in restaurants, customers have no effective way of predicting the number of calories in these dishes, which tend to be high in fat and low in nutritional content as well as in larger portions than people consume at home, which may negatively affect their public health. Localities across the US began enacting menu labelling regulations in 2008, beginning with New York City [26]. However, the results of such interventions tend to have varied in practice, with limited effects on pushing people to healthier options at a variety of impact sizes [27,28,29,30].

**Figure 1:**
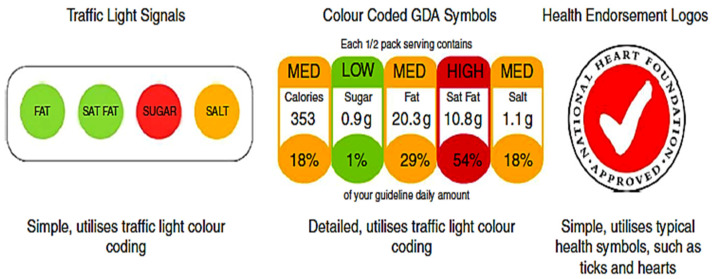
Popular nutrition labeling formats. Source: Montandon and Colli, [31].

**Figure 2:**
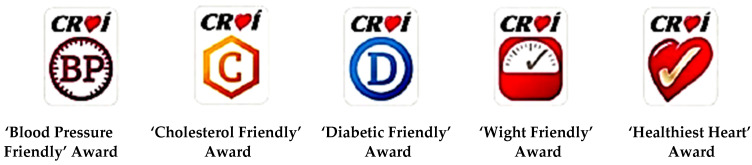
Icon-based menu labels. Source: Kerins et al., [32].

According to Scarborough et al. [31], nutrition labels consist of health endorsement logos, substantial nutritional information and even a simple “traffic light” method of affixing coloured nutritional symbols to food product packaging to signal the degree of healthiness are all examples of nutrition labelling styles (commonly used on ready-made meals in grocery stores throughout parts of Europe). Montandon and Colli [32] identified the most common nutrition-label formats for showing the nutritional information that customers of fast food restaurants were most familiar with (see Figure 1). Kerins et al. [33] designed icon-based menu labels (see Figure 2) based on the traffic light colour-coding system [34], which aims at improving outcomes through food and beverage services [35]. Authors of the same research [35] also suggested that future research should look into how individual differences in socio-demographics, health values and pre-existing nutrition knowledge may impact the effectiveness. According to Zhu [36], consumers would prefer to have the most information available, but they do not always use nutrition information to make purchase decisions. This is due to a variety of socio-demographic characteristics such as gender, income, education, family size, and so on. Furthermore, Green [37] argued that income had a substantial relationship with menu label utilisation, with higher-income consumers being more likely to use the information. Additionally, the same study [37] showed that more educated people had better public health outcomes in general, including lower risk of diabetes.

Most of the FFOs nowadays include nutrition information on their websites; however, it might be difficult to find; while making ordering decisions. Many American brands in Egypt, such as McDonald’s, have begun to provide nutrition labelling on their websites and with each served meal in various formats. Customers would prefer to have as much information as possible, but they do not always use nutrition information to make purchasing decisions, which is due to a variety of socio-demographic characteristics such as gender, income, education and family size [36]. With regard to the use of menu labels, there was a significant link between income and information use, with higher-income people being more likely to use it [37]. Additionally, more educated people have better health outcomes in general, including a lower risk of diabetes [37]. Studies on menu labelling have found a strong link between the target audience and the impact of nutrition labelling on consumer food choices. In this regard, a FFO menu calorie labelling study discovered a decrease in calories selected only by non-obese people [38]. On one hand, several research studies, see for example, [30,39], found that menu calorie labelling has only minor effects on food purchasing behaviour. On the other hand, various studies have found that menu labelling reduces the number of calories purchased [27,40], and others have even found no effect on consumer behaviour [28,29,41,42,43].

### 2.2. The Research Conceptual Model

The TPB theory outlines the factors that determine individuals’ intentions and hence their behaviours [44]. According to the TPB, the determinants of an individual’s intention are: attitude, subjective norms and perceived behavioural control. According to the theory, attitude refers to the extent to which an individual has a positive or negative assessment of the behaviour in question. Subjective norms are related to the perceived social influence to behave in a certain way. Perceived behavioural control is concerned with an individual’s perceptions of the ease or difficulty of behaving in a certain way; hence, it reflects previous experience and expected obstacles. The TPB confirms that that the only determinant of behaviour is intention. The TPB suggests that individuals with positive attitude, positive social influence and good perceived behavioural control are more likely to have the intention to perform a behaviour [44,45,46,47].

The TPB was adopted extensively to predict customer intention and behaviour in different contexts beyond the hospitality and tourism contexts. With regard to the hospitality context, the theory was adopted to predict food consumption decisions [17], intention to purchase local food products [18], customer intention and behaviour towards food waste [19], intention and behaviour towards menus with organic information in restaurants [20], attitude towards and intention to buy organic food [9], intentions to select eco-friendly restaurants [48] and purchasing intentions of genetically modified foods based on their nutrition labelling [49]. Recent research [46] suggested that attitudes, descriptive norms and nutrition literacy are significant predictors of food label use intentions. Moreover, perceived behavioural control (PBC) and reading ability are significant predictors of food label usage. These studies are some examples of the plethora of research adopting the TPB to predict customer intention and behaviour.

**Figure 3:**
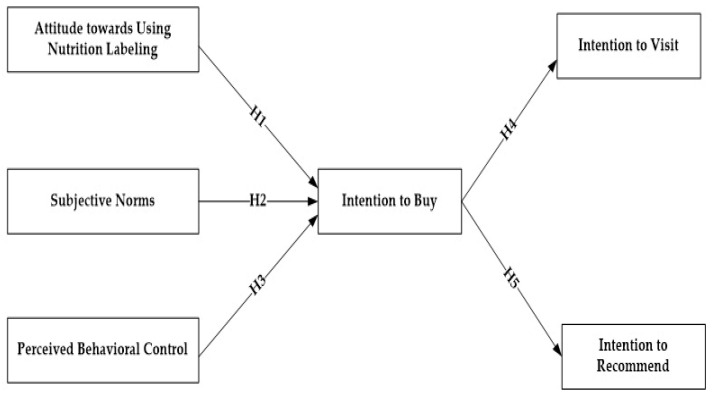
The proposed research model.

This research draws on the TPB to examine customer attitudes and behaviours related to buying food from FFO menus with nutrition labelling. In the research conceptual model (see Figure 3), a person’s intention to choose items with nutrition labels is formulated by their attitudes, subjective norms and perceived behavioural control. The TPB supports this notion that customers’ intention to choose food menus with nutrition labelling is positively influenced by these three constructs of TPB and to predict their buying behaviour. Thus, the study hypothesis are as follows:

**Hypothesis** **1** **(H1):** *Attitude toward nutrition labelling positively affects customers’ intention to buy nutrition-labelled items.*

**Hypothesis** **2** **(H2):** *Subjective norms positively affect customers’ intention to buy nutrition-labelled items.*

**Hypothesis** **3** **(H3):** *Perceived behavioural control positively affects customers’ intention to buy nutrition-labelled items.*

**Hypothesis** **4** **(H4):** *Customers’ intention to buy positively affects customers’ intention to visit restaurants with nutrition-labelled items.*

**Hypothesis** **5** **(H5):** *Customers’ intention to buy positively affects customers’ intention to recommend restaurants with nutrition-labelled items.*

## 3. Methodology

### 3.1. Data Collection

The study originated from a need to understand customers’ buying intentions and behaviours in the context of FFO food menus with nutrition labelling. Therefore, the study adopted a pre-tested questionnaire. A total of 600 questionnaire forms were distributed to random samples of FFO customers. The random samples of customers were at McDonald’s and Subway restaurants in Cairo and Giza, Egypt. The managers of the restaurants were asked to approach their customers and ask them to participate in the study. Managers agreed to approach their customers and obtain their consent to participate. Customers were asked to voluntarily participate in the research study. Written consent was obtained from each respondent before the data collection. The questionnaire forms were collected with the support of a company that specialized in data collection. Among the distributed forms, only 408 of the forms were completed and valid for data analysis, which represents a 68% response rate. This number of respondents was a sufficient sample size according to Krejcie and Morgan [50], who argued that the sample size should be 384 or above for a population of one million and above. This research sample also compared favourably with similar research sample sizes [20]. The distribution and collection of the questionnaires took nearly three months (December 2021 until February 2022).

### 3.2. The Research Questionnaire

The TPB variables (including intention and behaviour) were measured using a 20-item scale that was adopted from [20,51,52,53] as follows: attitude (AT), subjective norms (SN), perceived behavioural control (PBC), intention to buy, intention to visit, and intention to recommend. In order to enhance the response rate [54], items were pre-tested to make the questionnaire as short as possible. Furthermore, only important demographic information was collected. In addition, a high response rate was ensured due to the support of a specialized data collection company. Furthermore, respondents were asked to indicate their agreement on 5-point Likert scales ranging from (1) “strongly disagree” to (5) “strongly agree” instead of 7-point or 10-point Likert scales; 5-point scales require less time, are easy to answer [55] and help yield data sets that are amenable to advanced parametric and multivariate statistical analysis [56].

### 3.3. Data Analysis Methods

The current study screened the data and employed the following methods of analysis. First, the analysis of the preliminary data such as calculating means, standard deviations and outliers was conducted using statistical package for social sciences (SPSS v.25). Second, the model of this study containing the effects of the three constructs of the TPB on intention and ultimately on customer behaviour of visiting and recommending restaurants based on nutrition label provided was examined via structural equation modelling (SEM) using the analysis of moment structure (AMOS) version 23.

## 4. Results

### 4.1. Demographic Data Analysis

Questions related to the respondents’ demographic data asked about their age, gender, education level and diet status (see Table 1). As Table 1 shows, the majority of respondents, 67.6%, were in the age range from 21 to less than 30 years, followed by the respondents from 30 to less than 40 years, with a percentage of 12.3%. Moreover, 17% of the respondents were under 21 years (only those 18 or above could participate in the current study; hence, no children participated in this research). Only 2.9% of the respondents were from 40 to 50 years old, which reflects that young people (often 40 years or younger) are the dominant segment of FFO customers in Egypt. The sample showed similar proportions of female (50.8%) and male (49.2%) respondents. Concerning the level of education, the distribution was obviously skewed toward the highly educated sector of the population, with 66.6% of the sample having completed a bachelor’s degree, and 31.9% of the sample having completed a master’s or a doctoral degree. Only 1.5 of the respondents were secondary school students. Regarding diet status, only 28 (14.2%) of the respondents were following a special diet; most were not (71.6%).

### 4.2. Confirmatory Factor Analysis

The research adopted a confirmatory factor analysis (CFA) to verify the factors of the scale to be used for the data collection. The results from the CFA model showed that the model has good fit indices χ^2^ (32, *n* = 408) = 107,760, *p* < 0.001, normed χ^2^ = 3.367, SRMR = 0.04233, CFI = 0.981, TLI = 0.966, NFI = 0.932, PCFI = 0.771, and PNFI = 0.669). These fit indices confirm that the measurement model produces the data collected. For example, as [57] suggested, the value of the normed χ^2^ must be less than 5, and in this study, it was χ^2^ = 3.367. Additionally, the value of RMSEA must be less than 0.08 according to Pedhazur and Schmelkin [58], and here, it was 0.062. The incremental fit indices, NFI, TLI, and CFI, have to reach a threshold value of 0.90 [59], and these values were excellent in the current research (see Table 2). Furthermore, the results (see Table 2) for the skewness and kurtosis coefficients confirm that the study did not violate the assumption of normality.

Convergent validity was adopted to ensure that the variables correlated and could measure the phenomenon. Bentler and Bonett [60] stated that the composite reliability (CR) must be above 0.7 and the average variance extracted (AVE) above 0.5. As Table 3 and Table 4 show, all the values (CR, AVE and discriminant validity) in this research meet the required standards according to [60]. Discriminant validity was tested with a correlation matrix, the square roots of the AVEs and Cronbach’s alpha for each variable. Following the guidelines of Fornell and Larcker [61], discriminant validity was confirmed in this research (see Table 3). All Cronbach’s alphas were excellent or acceptable as recommended in previous research [62,63] since they all exceeded 0.7 (between 0.75 and 0.91; see Table 4).

### 4.3. Structural Equation Modelling Results

**Figure 4:**
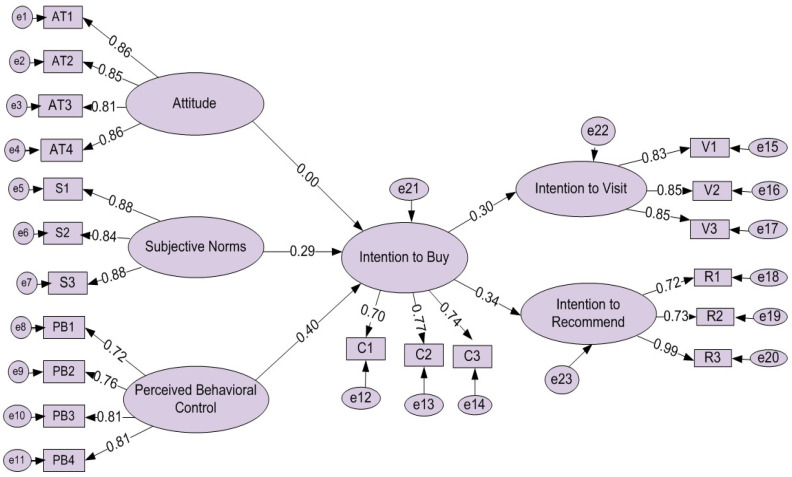
The final research structural model.

As highlighted above, structural equation modelling (SEM) was adopted to test the impacts of nutrition labelling on FFO customer buying intentions and behaviours using the TPB. The SEM results (see Table 4 and Figure 2) show that the structural model has good fit (χ^2^ (27, *n* = 408) = 74,520, *p* < 0.001, normed χ^2^ = 2760, RMSEA = 0.057, RMR = 0.059, SRMR = 0.038, GFI = 0.910, CFI = 0.979, TLI = 0.939, NFI = 0.889, PCFI = 0.718 and PNFI = 0.809). The results supported all research hypotheses except H1, regarding which, the study did not confirm a positive significant influence of attitude on intention to buy items with nutrition labelling (see Table 5). The results showed a positive significant influence of subjective norms on customers’ intention to buy (β = 0.201, t-value = 23.081, *p* < 0.001). Moreover, the results of the SEM model confirmed a positive direct path form perceived behavioural control to customers’ intention to buy (β = 0.297, t-value = 13.481, *p* < 0.001) and from customers’ intention to buy to their intention to visit (β = 0.411, t-value = 5.493, *p* < 0.001). Additionally, the results confirmed a positive direct influence of customers’ intention to buy on customers’ intention to recommend (β = 0.453, t-value = 6.325, *p* < 0.001) (see Table 5 and Figure 4).

## 5. Discussion and Implications

This study focused on customers’ buying intention and behaviours towards using nutrition labelling in FFOs in Egypt. Although most customers in this study were not on a special diet, they had high intentions and behaviours regarding buying nutrition-labelled items in FFOs in Egypt. The results revealed the presence of positive and direct significant paths from the constructs of the TPB, except for that from customers’ attitude to their buying intention. The results demonstrated no significant path from attitude to customer intention to buy, and thus, hypothesis one (H1) is not supported. This result does not support the work of Syed and Nazura [64], who found a positive significant influence of attitude on food buying intention. However, their research focused on halal food, which is an important cultural (mostly religious) element influencing attitudes and purchasing behaviour. Notwithstanding, the issue of healthy eating is not deeply related to culture. Moreover, most of the respondents were not on special diets and had not assessed their own attitudes towards using nutrition-labelled items. Although most respondents were highly educated, they paid little attention to special diets and to their attitudes towards the usage of nutrition labelling in FFOs. Hence, their attitudes did not have a significant influence on their buying intention and behaviours. In one comparative international study [65], Egyptian customers viewed McDonald’s more favourably than customers in other countries such as the US, where customers viewed it more critically. Egyptian customers often consume fast food, especially from international brands, without full attention to the type of food served or its calories provided. Again, this could justify the finding here of no statistically significant influence on respondents’ intention to use items with nutrition labels in FFOs.

The impacts of subjective norms on customer intention to buy were positive and statistically significant as was hypothesized. This finding supported hypothesis two (H2). This finding concurs with the work of Shin et al. [20], who found that customers’ buying intentions for specific food types were influenced by subjective norms. Egypt is classified as a collective society according to Hofstede [66]; hence, unsurprisingly, the results of previous research [65] have confirmed that Egyptian customers are influenced by their friends and families and thus spend much time at McDonald’s together. This research showed that respondents are influenced by their peers, even if they are minorities, to use the nutrition labelling provided at McDonald’s and other international restaurants.

The results revealed the presence of positive and significant path from perceived behavioural control to intention to buy supporting hypothesis three (H3). This result is consistent with previous studies [9,49,67], which also found a positive significant influence of perceived behavioural control on customers’ intentions to buy nutrition-labelled items. This means that respondents were confident and had the time and money to buy nutrition-labelled items when these was offered to them at FFOs; hence, this positively affected their intention to buy items with nutrition labels at FFOs. This could because most respondents were highly educated and had more control of their time and money; previous research studies [37] showed that educated people are more likely than others to control their resources (money and time) and buy healthier foods than others.

The results revealed a positive and significant influence of intention to buy on respondents’ intention to visit and on the path from intention to buy to intention to recommend, supporting hypotheses four (H4) and five (H5), respectively. These results are consistent with other studies [67,68] that also found that that the TPB constructs successfully predicted customers’ intention and ultimately their behaviour towards not just food choices but also towards revisit and recommendations to others [20]. This research showed that respondents have the intention to revisit these FFOs with nutrition-labelled menus and recommend them to their networks.

These research findings have some implication for hospitality scholars, especially those related to FFOs. The research adopted TPB to gain a better understating of customers buying intention and behaviours towards FFOs featuring nutrition labelled items. This research highlighted the role of both perceived behavioural control and subjective norms in buying items with nutrition labelled in FFOs. This research showed that customers’ attitudes were not a significant factor in their use of nutrition labels; they are more affected by their friends and families (subjective norms). The TPB explains why customers intend to buy nutrition-labelled items from FFOs. Based upon these results, there are also some managerial implications for those in FFOs. One of the most important issues that top management of these restaurants should consider is media campaigns to influence customer attitudes towards the use of nutrition information and labels to make healthy food choices at FFOs. These campaigns should reach potential customers through different methods including social media sites. Executive managers should be keen to introduce clear, coloured and briefly detailed nutrition labels for their customers at their purchasing units to drive their buying intention and behaviour towards ordering menu items with nutrition labelling and encourage healthy food choices. Calories, fat, cholesterol and saturated fat appeared to be the four nutrients perceived by customers as the most important for including on the menu [6]. It is also recommended that the top management of FFOs consider providing a variety of nutrition label formats in order to meet the demands of different customer segments for healthier choices. Additionally, FFOs should empower service staff by giving them training courses that enable them in becoming knowledgeable about such nutrition issues in order to help customers make the right food choices. FFO marketers should offer advertising and marketing strategies based on customer preferences regarding indoor nutrition labelling and online nutrition information. Policymakers, on the other side, should enact effective rules and legislation to help FFO customers avoid obesity, overweight and related issues that results from making unhealthy choices at FFOs that do not provide nutrition labelling to promote proper public health.

## 6. Conclusions

This study examined Egyptian customers’ intention to buy fast food items with nutrition labelling at two FFOs. Additionally, the research examined the influence of nutrition labelling on customers’ intentions to visit and recommend these FFOs. The study draws on the TPB to properly understand and examine customers’ intentions to buy nutrition-labelled menus and their behaviour of visiting and recommending these FFOs. The conclusions of the current research are as follows: The research confirmed the positive and significant influences of subjective norms and perceived behavioural control on customer intention to buy items with nutrition labels at FFOs. However, unlike previous research studies, the current research did not confirm a direct positive influence of attitude on customer intention to buy items with nutrition labels. This could be because most respondents were not on special diets and had no assessments of their attitudes towards using nutrition-labelled items. Furthermore, the current research extended the TPB research framework and showed that customer intention to buy menu items from FFOs that provide nutrition labels has a direct positive influence on their revisit intentions and recommendations of these FFOs to peers, friends and networks.

## 7. Limitations of the Study and Further Research Opportunities

The current research has drawn on the TPB to examine the role of nutrition labelling in buying intention and behaviour at FFOs in a sample of one single market, Egypt. Additionally, the research adopted a questionnaire survey, which has some limitations as a data collection tool. Hence, the results cannot be simply generalized to other country contexts without further examination. This research did not examine the role of culture in customer choices of food based on their nutrition labels. Future researchers could examine this issue by adopting an international comparative study between counties with different cultural dimensions to examine whether culture moderates customer choices of health foods.

## Figures and Tables

**Table 1:** The respondent profile.

Profile		Freq.	%
Age	Under 21 Years	50	12.3
	From 21 to under 30 Years	276	67.6
	From 30 to under 40 Years	70	17.2
	From 40 to under 50 Years	12	2.9
	50 Years and Over	0	0
Gender	Male	201	49.2
	Female	207	50.8
Level of Education	Secondary School Diploma or less	6	1.5
	Bachelor’s Degree	272	66.6
	Master’s Degree	88	21.6
	Doctoral Degree or Equivalent	42	10.3
	Other	0	0
Diet Status	Not on special diet	292	71.6
	On special diet	58	14.2
	Low fat	28	6.8
	Low sodium	0	0
	Low calorie	24	5.9
	Vegetarian	6	1.5
	Others	0	0

**Table 2:** The descriptive statistics for the research items.

Abrev.	Item	Min	Max	M	SD	Skewness	Kurtosis
Attitude							
AT1	Using nutrition labelling is an advantageous action.	1	5	4.06	0.896	−0.811	0.365
AT2	Using nutrition labelling is a wise action.	2	5	4.11	0.865	−0.604	−0.507
AT3	Using nutrition labelling is a pleasant action.	2	5	4.18	0.907	−0.881	−0.105
AT4	Using nutrition labelling is an attractive action.	1	5	3.99	0.968	−0.775	−0.037
Subjective Norms							
S1	People who are important to me think I should choose a nutrition-labelled item when eating out.	2	5	4.31	0.783	−0.856	−0.078
S2	Most people who are important to me would want me to choose a nutrition-labelled menu item when eating out.	1	5	3.99	0.968	−0.775	−0.037
S3	People whose opinions I value would prefer me to choose a nutrition-labelled item when eating out.	2	5	4.20	0.818	−0.746	−0.155
Perceived Behavioural Control							
PB1	I am confident that if I want, I can choose a nutrition-labelled item when eating out.	2	5	4.18	0.820	−0.548	−0.723
PB2	I am capable of choosing a nutrition-labelled item when eating out.	2	5	4.31	0.783	−0.856	−0.078
PB3	I have enough resources (money) to choose a nutrition-labelled item when eating out.	2	5	4.11	0.847	−0.568	−0.536
PB4	I have enough time to choose a nutrition-labelled item when eating out.	2	5	4.03	0.826	−0.444	−0.533
Intention to Buy							
C1	I am planning to choose a nutrition-labelled menu item when eating out in the future.	2	5	4.07	0.837	−0.410	−0.816
C2	I intend to choose a nutrition-labelled menu item when eating out in the future.	2	5	4.18	0.820	−0.548	−0.723
C3	I will expend effort on choosing a nutrition-labelled menu item when eating out in the future.	2	5	4.31	0.783	−0.856	−0.078
Intention to Visit							
V1	I am planning to visit a restaurant featuring nutrition labelling in the future.	2	5	4.11	0.847	−0.568	−0.536
V2	I intend to visit a restaurant featuring nutrition labelling in the future.	1	5	4.06	0.896	−0.811	0.365
V3	I will expend effort on visiting a restaurant featuring nutrition labelling in the future.	2	5	4.11	0.865	−0.604	−0.507
Intention to Recommend							
R1	I am planning to recommend a restaurant featuring nutrition labelling when someone asks me about eating out in the future.	2	5	4.18	0.907	−0.881	−0.105
R2	I intend to recommend a restaurant featuring nutrition labelling when someone asks me for eating out in the future.	1	5	3.99	0.968	−0.775	−0.037
R3	I will expend effort on persuading everybody who asks me about eating out to visit a restaurant featuring nutrition labelling in the future.	2	5	4.18	0.820	−0.548	−0.723

Model fit: (χ^2^ (32, *n* = 408) = 107,760 *p* < 0.001, normed χ^2^ = 3.367, RMSEA = 0.062, SRMR = 0.04233, CFI = 0.981, TLI = 0.966, NFI = 0.932, PCFI = 0.771 and PNFI = 0.669), Note: Min = minimum, Max = maximum, M = mean, SD = standard deviation.

**Table 3:** The convergent validity results.

Factors and items	FL	CR	AVE *	MSV **	ASV ***
Attitude		0.880	0.648	0.402	0.389
- Using nutrition labelling is an advantageous action.	0.861				
- Using nutrition labelling is a wise action.	0.850				
- Using nutrition labelling is a pleasant action.	0.814				
- Using nutrition labelling is an attractive action.	0.862				
Subjective Norms		0.900	0.751	0.650	0.624
- People who are important to me think I should choose a nutrition-labelled item when eating out.	0.878				
- Most people who are important to me would want me to choose a nutrition-labelled menu item when eating out.	0.842				
- People whose opinions I value would prefer me to choose a nutrition-labelled item when eating out.	0.879				
Perceived Behavioural Control		0.858	0.603	0.538	0.480
- I am confident that if I want, I can choose a nutrition-labelled item when eating out.	0.721				
- I am capable of choosing a nutrition-labelled item when eating out.	0.761				
- I have enough resources (money) to choose a nutrition-labelled item when eating out.	0.812				
- I have enough time to choose a nutrition-labelled item when eating out.	0.806				
Intention to Buy		0.782	0.545	0.306	0.298
- I am planning to choose a nutrition-labelled menu item when eating out in the future.	0.701				
- I intend to choose a nutrition-labelled menu item when eating out in the future.	0.772				
- I will expend effort on choosing a nutrition-labelled menu item when eating out in the future.	0.740				
Intention to Visit		0.882	0.714	0.193	0.147
- I am planning to visit a restaurant featuring nutrition labelling in the future.	0.832				
- I intend to visit a restaurant featuring nutrition labelling in the future.	0.851				
- I will expend effort on visiting a restaurant featuring nutrition labelling in the future.	0.852				
Intention to Recommend		0.862	0.682	0.205	0.202
- I am planning to recommend a restaurant featuring nutrition labelling when someone asks me about eating out in the future.	0.724				
- I intend to recommend a restaurant featuring nutrition labelling when someone asks me about eating out in the future.	0.727				
- I will expend effort on persuading everybody who asks me about eating out to visit a restaurant featuring nutrition labelling in the future.	0.996				

* AVE = Average Variance Extracted; ** MSV = Maximum Shared Value, *** ASV = Average Shared Value.

**Table 4:** The discriminant validity results.

Factors	1	2	3	4	5	6
1-Attitude	0.805 *					
2-Subjective Norms	0.480	0.867 *				
3-Perceived Behavioural Control	0.056	0.528	0.776 *			
4-Intention to Buy	0.168	0.668	0.683	0.738 *		
5-Intention to Visit	0.798	0.375	0.413	0.396	0.850 *	
6-Intention to Recommend	0.767	0.664	0.335	0.445	0.555	0.825 *

* Note: the values on bold represent the square root of average variance extracted (AVEs).

**Table 5:** The results for the structural model.

Hypotheses			β	C-R T-Value	Results
H1- Attitude	→	Intention to buy	NS	0.022	Not supported
H2- Subjective norms	→	Intention to buy	0.201 ***	23,081	Supported
H3- Perceived behavioural control	→	Intention to buy	0.297 ***	13,481	Supported
H4- Intention to buy	→	Intention to visit	0.411 ***	5493	Supported
H5- Intention to buy	→	Intention to recommend	0.453 ***	6325	Supported

Model fit: (χ^2^ (27, *n* = 308) = 74,520 *p* < 0.001, normed χ^2^ = 2760, RMSEA = 0.057, RMR = 0.059, SRMR = 0.038, GFI = 0.910, CFI = 0.979, TLI = 0.939, NFI = 0.889, PCFI = 0.718 and PNFI = 0.809), *** *p* < 0.001.

## Data Availability

Data are available upon request from researchers who meet the eligibility criteria. Kindly contact the first author privately by e-mail.
